# Supplementary material for: Study protocol for transforming health equity research in integrated primary care: Antiracism as a disruptive innovation
Source: PLoS One. 2024 Jun 27;19(6):e0306185. doi: 10.1371/journal.pone.0306185 (PMC11210870; doi:10.1371/journal.pone.0306185)
Supplement: Attachment — Submitted filename: Naar et al review.pdf [file pone.0306185.s001.pdf]

Naar et al. review

Major comments:

- This is exciting research and I applaud the authors for addressing racism directly. I look forward to seeing the results of this ambitious research!
- Line 172: The paper goes directly from aims to the ethical considerations section, which mentions a lot of activities (e.g., GMB sessions). It seems like a description of these activities directly following the aims and prior to the ethical considerations section would be helpful for the reader. The advisory councils should be introduced here.
- I wonder if the participants / recruitment section should go after the description of group modeling (starting line 255), so the reader knows what the recruitment is for.
- Line 289: Until this mention of agents, I had assumed that your reference to computational simulation modeling referred to system dynamics modeling. But it seems like you're using agent-based simulation? I think this should be more clearly stated earlier in the methods (and likely the intro and abstract). Using ABM alongside systems mapping in a GMB process is not as common as SD modeling and represents a unique aspect of this study.
- I was surprised to see a results section in a protocol paper, and also a bit confused about its contents. Please see minor comments below for questions re: timing, etc. I think you have a judgement call to make regarding whether the protocol should describe your original plans only or whether you'd like to include the additions / changes you made that are described now in the results. If you keep the additions / changes, I'm wondering if it would be clearer for the reader if the protocol was written as if all activities were proposed and would happen in the future. The detail about changing the stakeholder groups and engagement due to adapting to feedback could be detailed in future publications when communicating study results. But this is ultimately up to the authors and the journal editors. If the results section is kept, I'd recommend adding some explanatory text earlier so the reader understands what to expect regarding timing and changes. I feel like I'm not communicating this concern very well – please feel free to reach out if it's not clear.
- Line 405: Somewhere, the paper would benefit from a clearer description of these theories and how they were integrated into your approach. Also, should item response theory be included?
- Discussion: A few questions I'd like to see addressed in the discussion (or somewhere in the text):
    - What are the mechanisms by which the modeling activities will lead to change for clinics and patients? Are you expecting participants to change their own behavior or the policies / procedures at their clinics or in their communities? How will you know change has happened?
    - Engaging in modeling is time intensive. How will you ensure that this experience is worthwhile for participants and not an additional burden? Is it safe to assume that people who participate are likely to be people concerned about addressing racism? If that's the case, how will you avoid 'preaching to the choir' to ensure that structural changes actually get identified / made?

- Simulation requires data or assumptions to provide a foundation. I'm guessing that this model will require a lot of expert judgement due to a lack of extensive data. Building simulation models based on expert / stakeholder judgement is a valid approach, but necessarily involves challenges regarding how to elicit and combine perspectives into a single model. I'd like to see the authors address how they'll approach these challenges in the methods and then discuss the broader implications (e.g., validity, generalizability, etc.) in the discussion.

Minor comments:

- The paper would benefit from minor proofreading corrections and grammatical edits (e.g., in line 71, 'is' should be 'are'; the quotation starting in line 77 is not ended).
- Line 127: Is ORBIT an acronym? If so, spell it out. This section would also benefit from more description of the ORBIT model (e.g., how many phases does it have, is it iterative, what purpose does it serve, etc.) prior to discussing the individual phases. A figure or table summarizing this model might also be helpful.
- Line 147: I recommend rewording the reference to the figure to make it clear that the figure shows the protocol, which draws from the ORBIT model. The text now seems to say that the figure summarizes the established model.
- Line 157: I recommend having some kind of introductory text at the beginning of the methods giving a broad summary of the methods to be used, and maybe some introductory text prior to the list of methods to set them up. I also recommend either rewording the list of aims so that they are in full sentences or reformatting into a table with aim, approach, etc.
- Line 169: Either here or earlier, a description of item response theory would be helpful.
- Line 201: At first I thought this section missed info about recruitment, but then I saw the following subsections. You might want to have a sentence in this first section that refers to different recruitment strategies for different stakeholder types to cue up the subsections. Also, are participants assembled into an expert panel? Are they reimbursed? How will it be determined whether participants identify as Black/African American?
- Line 203: Be consistent about past vs future tense throughout.
- Line 223: How will patients be contacted?
- Line 231: I'm a little confused about the different groups. You refer to recruiting participants, having community advisory groups (line 225) and now scientific advisory councils. A high level overview of the different groups or ways that participants interact with this research would be helpful, preferably at the beginning of the methods section.
- Line 255 section: Will patients and stakeholders from other groups participate in causal mapping together or separately? How will you handle potentially different perspectives between individuals and groups? How are modeling decisions made? Will racism and other key concepts be defined previously or defined by the group(s)?
- Line 283 Simulation process description: How will stakeholders be involved in operationalizing the simulation model? Will they help choose key indicator variables or have those been chosen (and if so, how?)? Will there be some iteration between model drafts and participant feedback?

Will any quantitative data be used as a basis for model parameterization or will it be based solely on expert opinion?

- Line 332: Which initial activities? I had thought this protocol proposed future activities? Or are you referring to preliminary research that served as a basis for this proposed research?
- Line 336: Similarly, I'm wondering here about the timeline – Did recruitment already happen for these councils? It might be simpler for the reader if the protocol was worded in the future tense and the people's health justice committee is described alongside the other councils.
- Figure 2: The text in the figure is very small and will be difficult to read in the article pdf. Can this be revised to be more legible?
- Line 428: The community engaged dissemination plans should be better described prior to the discussion.
