# Supplementary material for: Study protocol for transforming health equity research in integrated primary care: Antiracism as a disruptive innovation
Source: PLoS One. 2024 Jun 27;19(6):e0306185. doi: 10.1371/journal.pone.0306185 (PMC11210870; doi:10.1371/journal.pone.0306185)
Supplement: Attachment — Submitted filename: Revision.docx [file pone.0306185.s002.docx]

Abstract:

It took me a while to realize that this study is to assess Racism in the African American (AA) population.

Not only is it not mentioned in the Abstract, but it also needs to be clarified in the Intro (see my comments below). It is essential to be intentional about describing in the Introduction why you are doing this system-wide intervention and measuring it only amongst AA (leaving all the other rich Florida diversity).

Introduction:

Having the first phrases describing only AA statistics is confusing, particularly after the Abstract does not mention that you will only look at the AA community. I recommend starting with a broad description of the issue that affects the global majority (those racialized and historically marginalized communities, formerly known as minorities). Being that the funding goes to researchers in Florida, I am surprised that there are no reports of disparities amongst Latine and Immigrant communities. Make the case why, and probably given the need to involve the community; funding is limited to do this work in a participatory way with one. Build the case for that.

It is important to clarify that there are disparities in health co-morbidities and mental health due to Structural Issues (social determinants of health), which create the main difference between communities. Thus, many Community-based, policy-based structural interventions exist to stop that. Then there is a different set of differential treatments that happens when a patient comes (if they can) to our system: unequal treatment. This is what you are referring to, and this is what is essential to highlight. Even when we put office-based interventions to buffer some of the SDOH impacts, it will differ from the band-aid we are creating locally. Ensure some of this goes into the Introduction, explaining the disparities' origin. See the figure below; you have this citation as Number 1.

In the Introduction, I advise discussing concepts about Intersectionality and how they play a role in the pile of discrimination and how we need to account for that when talking about anti-oppression. There are several articles defining the need for this. One has coined the name of Integrative racism (Nelson, L.E., Reeves, J. and Lopez, D.J., 2023. Integrative Antiracism and the Salience of Intersectional Assets for Black and Latinx LGBTQ Youth. *Journal of Adolescent Health*, *72*(5), pp.647-648.), to signal that we need to pay attention to the interaction of other marginalized identities when designing anti-oppressive interventions, even when we are trying to tackle the forgotten one: racism.

Line 161: Add African American to "Community-engaged Group Model Building (GMB)"

Line 220: are the stakeholders paid? A genuinely participatory community-based study pays the stakeholders, patients, and providers for their involvement and time. I hope you do, and if so, add it to the protocol. Explain how you are handling the payment (cards?). Same with CAC: are they paid?

Line 247 Define MPIs.

Line 291: you could add some neighborhood-level data as part of contextual data when selecting patients, as this needed to be included to "diversify" patients. Examples below:

Line 338: Hurray! I was going to ask why the Intro did not talk about CBPR, as this was what you were trying to do in the initial phase; I'm glad you figured that out.

Line 346: paid? This is one of the most essential parts of a participatory process. If you are not doing that now, re-think how you could do that.

349: Excellent!

Overall result-discussion: I will probably eliminate the section Results (you mentioned before in the abstract that you would not share results, which are not results). I would incorporate this into the Protocol in Orbit phase 0. You could clarify there that this was not an initial component of the Funding (it seems like it was not), but for readers to understand the protocol, I will include it there, with a little summary on how you learned the importance. At this point in science, it is well established that this participatory component is one of the most important in Health Equity science and probably was an oversight on your initiative. I will add a paragraph about CBPR and discuss why. It seems more straightforward and accessible for the reader for protocol publishing purposes. This is my recommendation, of course, it is up to you to decide. Then, you should leave the discussion as it is.

I will add somewhere in the translational piece that the researchers will engage with the Site Leadership to start working on their buy-in to incorporate the suggested lessons learned during this study. Leadership buy-in is one of the most critical components for sustainability and health equity transformation. Thanks for the fantastic work and effort to advance health equity!

Thanks again.
